# Serum levels of vitamin E, vitamin D, and omega-3 fatty acids in patients with generalized chronic periodontitis: A cross-sectional study

**DOI:** 10.34172/joddd.025.42534

**Published:** 2025-12-31

**Authors:** Shima Ghasemi, Amirreza Babaloo, Mohammadtaghi Chitsazi, Atiyeh Ghassemi, Saeid Forghani

**Affiliations:** ^1^Department of Prosthodontics, Faculty of Dentistry, Tabriz University of Medical Sciences, Tabriz, Iran; ^2^Department of Periodontics, Faculty of Dentistry, Tabriz University of Medical Sciences, Tabriz, Iran; ^3^Pediatric Critical Care Fellow, Department of Pediatrics, Tabriz University of Medical Sciences, Tabriz, Iran

**Keywords:** Chronic periodontitis, Fatty acids, Omega-3, Vitamin D, Vitamin E

## Abstract

**Background.:**

The present study aimed to investigate the serum levels of vitamin D, vitamin E, and omega-3 in patients with chronic periodontitis.

**Methods.:**

Thirty-six patients with periodontitis participated in this cross-sectional descriptive study. Clinical parameters such as the gingival index, pocket depth (PD) (mm), and clinical attachment loss (CAL) (mm) were measured. Serum samples were obtained and analyzed for levels of vitamin D, vitamin E, and omega-3 fatty acids. The data were analyzed using SPSS 24, and a probability value of<0.05 was considered statistically significant.

**Results.:**

All the clinical parameters used to measure periodontal status differed significantly according to the severity of periodontitis (*P*<0.05). The mean serum levels of all vitamins and omega-3 fatty acids were lower in the severe periodontitis group than in the moderate periodontitis group. The mean levels were high in the mild periodontitis group. However, the differences were significant only for vitamin E and vitamin D (*P*<0.05). No statistically significant effect was observed for omega-3 fatty acids, but higher amounts of omega-3 fatty acids were detected in mild periodontitis patients than in moderate and severe periodontitis patients.

**Conclusion.:**

It can be concluded that the optimal consumption of vitamin E and vitamin D, either as supplements or as part of an individual’s daily diet, may contribute to maintaining periodontal health.

## Introduction

 Periodontitis is a prevalent chronic inflammatory disease characterized by the destruction of tooth-supporting tissues, including the periodontal ligament and alveolar bone.^1^ Chronic periodontitis is the most common form of periodontitis and is generally considered a slowly progressing disease. However, in the presence of systemic or environmental factors that change the host’s response to plaque accumulation, such as diabetes, smoking, and stress, disease progression is accelerated.^2,3^

 In recent years, increasing attention has been paid to lifestyle factors, particularly nutrition, as modulators of periodontal health.^4,5^ Healthy and appropriate nutrition is one of the main factors of oral health. Establishing proper eating habits not only improves physical growth and development but also provides a favorable environment for optimal oral health. Nutrient deficiencies may not directly initiate gingivitis, but they can exacerbate periodontal inflammation and tissue destruction.^6^ Nutritional poverty can cause changes in the primary factors of the etiology of periodontal diseases, whereas diet affects the progression of periodontal lesions.^7^ Studies have shown that dietary disorders are not initiators of gingivitis but can aggravate the disease. The onset and severity of gingival and periodontal disease are related to local stimulation factors, and a lack of nutrients increases the adverse effects of these factors.^8^

 As an essential nutrient, vitamin D plays a crucial role in the management of various inflammatory diseases. The active form of vitamin D, i.e., 1,25-dihydroxyvitamin D or 1,25(OH)_2_ D, is the main component involved in the process of regulating bone metabolism through increased absorption of calcium and phosphate.^9^ In addition, vitamin D modulates innate and adaptive immune responses, and its deficiency has been linked to greater periodontal attachment loss.^10^ Various studies have investigated the role of vitamin D supplementation in patients with moderate to severe periodontitis and have shown a positive effect on clinical parameters.^8,11,12^

 Like vitamin D, vitamin E also plays a pivotal role in preventing periodontal diseases. The primary role of vitamin E is its antioxidant function.^13^ The effect of vitamin E in periodontal diseases is related to its effects on free radicals and inflammation and its regulatory function in the immune system. Evidence suggests that vitamin E supplementation improves oxidative balance and reduces clinical signs of inflammation in periodontal patients.^14^

 Another effective nutrient is omega-3 fatty acids. Omega-3 fatty acids have beneficial anti-inflammatory effects on many organs, gums, and periodontal tissue. Through the production of specialized pro-resolving mediators such as “resolvins” and “protectins,” omega-3 polyunsaturated fatty acids can suppress cytokine production and neutrophil infiltration, thereby protecting periodontal tissues.^15^ Studies have shown that a diet containing omega-3 fatty acids improves the progression of periodontal diseases and reduces the prevalence of periodontitis.^16^

 To the best of our knowledge, few studies in Iran have addressed the serum levels of vitamin E, vitamin D, and omega-3 fatty acids in Iranian populations with generalized chronic periodontitis based on disease severity or limited scope, with a particular focus on patients.^17,18^ The present study investigated the serum levels of vitamin D, vitamin E, and omega-3 in patients with chronic periodontitis.

## Methods

###  Study Design and Setting

 We conducted a cross-sectional study in the outpatient clinic of the Faculty of Dentistry, Tabriz University of Medical Sciences, Tabriz, Iran, in 2022.

###  Sample Size

 We conducted a pilot study with eight patients with periodontitis. Four patients had mild periodontitis, and four had severe periodontitis. Power and sample size software was used to determine the sample size. In the pilot study, the sample size was estimated based on the standard deviation and mean values of vitamin D in two different severities of periodontitis. According to the following formula:

 n = (Z_1-α/2_ + Z_1-β_)^2^ × (σ_1_^2^ + σ_2_^2^)/(μ_1_ – μ_2_)^2^

 Considering α = 0.05, the study power was 80%, the standard deviation of group one was σ1 = 1.9, that of group two was 2 = 3.4, the mean for group one was μ1 = 12.5, that of group two was μ2 = 8, and an error of 10% was found for 12 individuals in each group (with three severities of periodontitis). The final sample size was estimated at 36 patients.

###  Inclusion and Exclusion Criteria

 Patients were included if they suffered from chronic periodontitis, had a loss of the interproximal clinical adhesion limit in at least 30% of the area, had bleeding during probing, or had at least 20 teeth. Pregnant or menopausal patients, patients with continuous use of medicines containing calcium, zinc, and iron in the last three months, patients with systemic disease, especially those with periodontal conditions such as diabetes, abnormalities of the immune system, and AIDS, or diseases that require antibiotic therapy, such as heart problems and joint replacement, were excluded. Additionally, drug use (patients who used NSAIDs, corticosteroids or have had antibiotics), receiving periodontal treatment in the previous year and scaling in the previous six months, smokers and alcoholics, patients with severe dental caries, and patients with chronic inflammatory diseases of the skin and oral mucosa (such as lichen planus, pemphigus, psoriasis, aphthous ulcers, and estrogen therapy) were exclusion criteria for this study.

###  Clinical Parameters

 The following clinical parameters were measured: gingival bleeding index, pocket depth (PD) (mm), and clinical attachment level (CAL) (mm). In the gingival bleeding index method, each tooth is probed gently by a periodontal probe at six probed locations, distal, middle, and mesial, on both the buccal and lingual surfaces, and bleeding is scored based on the presence or absence of bleeding and the number of bleeding sites.^19^

 Periodontal PD: The distance between the depth of the sulcus and the gingival margin of all existing teeth; the depth of the pocket in the mesiobuccal, midbuccal, distobuccal, mesiolingual, midlingual, and distolingual levels was measured via the Williams periodontal probe, and the fractional sizes were rounded to the nearest mm.^20^

 CAL: The distance between the depth of the sulcus and the cementoenamel junction (CEJ) of all existing teeth; CAL in the mesiobuccal, midbuccal, distobuccal, mesiolingual, midlingual and distolingual levels was measured via the Williams periodontal probe (hu-friedy, Chicago, IL, USA); and the measurements were rounded to the nearest mm.^20^

 The patients were classified into three groups,^21^ according to the CAL: mild chronic periodontitis with a CAL of 1–2 mm, moderate chronic periodontitis with a CAL of 3–4 mm, and severe chronic periodontitis with a CAL of > 5 mm.

###  Serum Analysis

 The patients were instructed to arrive early in the morning, fasting, to provide serum samples. In the laboratory, the serum sample was centrifuged at 4000*g for 8–10 minutes. The serum samples were stored at -20 °C until the tests were performed. Serum levels of vitamin D, vitamin E, and omega-3 fatty acids were measured using standard ELISA protocols, following the manufacturer’s instructions. The results were presented in terms of ng/mm.

###  Statistical Analysis

 Clinical parameters, including the mean gingival index, mean PD, and mean CAL, were calculated for each participant, along with the means and standard deviations (SDs) for each periodontitis severity group. Similarly, the means and standard deviations for the serum levels of micronutrients were also computed. Differences across the three severity groups were analyzed using one-way ANOVA, followed by post hoc Tukey tests where appropriate. All the data were compiled and analyzed via SPSS 24, and a *P* value of < 0.05 was considered significant. We adhered to the STROBE checklist for this study, which is attached as a supplement.

###  Ethical Considerations

 This study was approved by the Ethics Committee of Tabriz University of Medical Sciences (approval number: IR.TBZMED.REC.1402.134). In this study, the safety and health of the participants were observed. All the patients were informed about the objectives, benefits, and risks of participating in the study and signed an informed consent form before participating in the study. Patients were able to withdraw from the study at any time. Additionally, in this research, periodontitis was treated in patients.

## Results

###  Clinical Parameters

 Thirty-six patients (21 women and 15 men) with diffuse chronic periodontitis participated in this study. Twelve patients had mild periodontitis, 12 had moderate periodontitis, and 12 had severe periodontitis. Table 1 shows the comparison of clinically obtained data, including the gingival index, PD, and CAL, across different severities of periodontitis. There was a significant difference in the mean ages of patients with varying severities of periodontitis (*P* = 0.000). All the clinical parameters used to measure periodontal status significantly differed according to the severity of periodontitis (*P* < 0.05).

**Table 1:** Comparison of clinical parameters among the three periodontal groups

	**Mild periodontitis ** **(n=12)**	**Moderate periodontitis** **(n=12)**	**Severe periodontitis** **(n=12)**	***P *** **value**
Age (years) (mean ± SD)	34.83 ± 5.96	37.5 ± 8.78	58.25 ± 7.44	0.000^a^
Gingival bleeding index (mean ± SD)	0.42 ± 0.51	0.75 ± 0.45	1.00 ± 0.00	0.004^a^
Pocket depth (mean ± SD)	2.51 ± 0.62	5.13 ± 0.73	6.78 ± 0.67	< 0.001^a^
Clinical attachment level (mean ± SD)	1.34 ± 0.31	3.55 ± 0.48	5.72 ± 0.52	< 0.001^a^

^a^*P *value < 0.05 (significant).

###  Serum Vitamin Levels

**Figure 1:**
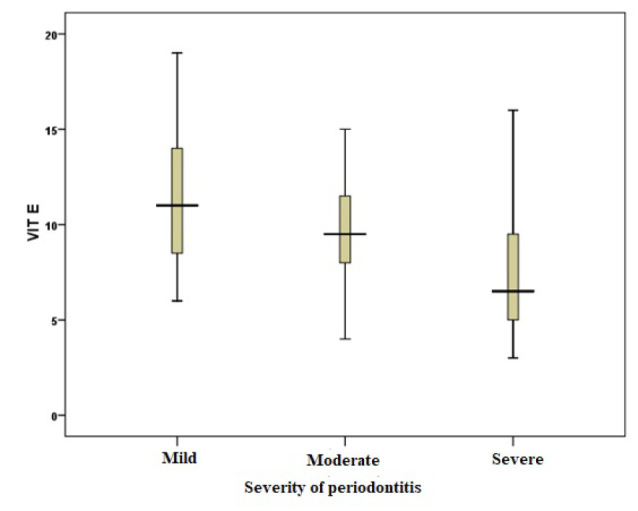
Comparison of vitamin E serum levels in terms of disease severity

**Figure 2:**
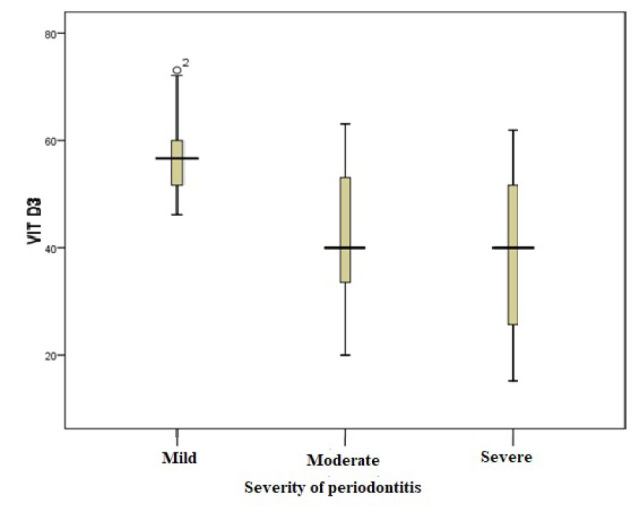
Comparison of vitamin D serum levels in terms of disease severity

**Figure 3:**
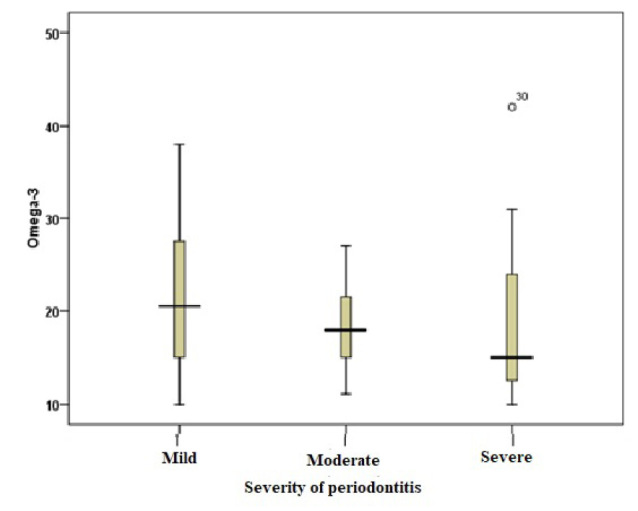
Comparison of omega-3 fatty acid serum levels in terms of disease severity

 The serum levels of vitamin E, vitamin D, and omega-3 fatty acids were analyzed in patients with different severities of periodontitis. The results are summarized in Table 2. The mean serum levels of all vitamins and omega-3 fatty acids were lower in the severe periodontitis group than in the moderate periodontitis group. The mean levels were high in the mild periodontitis group. However, the differences were significant only for vitamin E and vitamin D (*P* < 0.05) (Figures 1, 2, and 3).

**Table 2:** Comparison of serum vitamin levels

	**Mild periodontitis ** **(n=12)**	**Moderate periodontitis** **(n=12)**	**Severe periodontitis** **(n=12)**	***P *** **value**
Vitamin E	11.50 ± 3.97	9.67 ± 3.11	7.58 ± 3.63	< 0.001^a^
Vitamin D	57.33 ± 8.44	42.41 ± 13.09	38.33 ± 15.07	0.031^a^
Omega-3	21.83 ± 8.25	18.41 ± 4.75	18.91 ± 9.66	0.521

^a^*P* value < 0.05 (significant).

## Discussion

 This study investigated the serum levels of vitamins D and E, as well as omega-3 fatty acids, in patients with chronic periodontitis in relation to the severity of the disease. The patients in this study (36 individuals) were grouped according to the severity of periodontitis into one of three groups: mild, moderate, or severe periodontitis. Although significant associations were observed for vitamins D and E, no statistically significant association was detected for omega-3 fatty acids; however, higher amounts of omega-3 fatty acids were detected in mild periodontitis patients than in moderate and severe periodontitis patients, although this difference was not statistically significant.

 The present study revealed a significant association between serum vitamin E levels and the severity of periodontitis. The vitamin E levels in patients with mild periodontitis were significantly higher than those in patients with severe periodontitis. Behfarnia et al^22^ in a study on 16 patients with chronic periodontitis in two groups (with scaling and root planing [SRP] treatment with vitamin E consumption and without vitamin E consumption) reported that vitamin E supplementation with SRP could reduce the inflammatory process of periodontitis, improve periodontal clinical data, and reduce the amount of adhesion loss. According to a study by Shadisvaaran et al,^23^ vitamin E can improve periodontal conditions by correcting redox imbalance, reducing inflammatory responses and enhancing wound healing. However, strong evidence for the use of vitamin E supplementation or the treatment of periodontitis in humans is still limited, and robust studies are necessary to ensure its effectiveness. The results of the above studies are consistent with our findings of an association between higher vitamin E levels and milder periodontitis. Notably, vitamin E (α-tocopherol and related tocopherols/tocotrienols) stabilizes cell membranes by preventing lipid peroxidation, scavenges reactive oxygen species, modulates pro-inflammatory cytokine production, and can influence host antioxidant enzyme systems, pathways that plausibly reduce oxidative tissue damage and inflammation in periodontal lesions.

 On the other hand, Houshmand et al^24^ reported no significant additional benefit from the topical application of vitamin E in combination with SRP compared with SRP alone. The authors noted that improvements in clinical indicators were primarily influenced by time rather than the type of treatment. Differences in administration route, dosage, sample size, and follow-up duration may explain the variations in outcomes.

 The present study revealed a significant association between the mean serum levels of vitamin D and the severity of periodontitis. Vitamin D levels were significantly higher in patients with mild periodontitis and similar in patients with moderate and severe periodontitis. Consistent with the present study, higher serum levels of vitamin D have been reported in healthy patients or patients with mild periodontitis.^18,25,26^ According to the available evidence, in patients with treated periodontitis who were in the periodontal maintenance phase, compared with those in the placebo group, when vitamin D and calcium supplements were used, the values of probing depth, bleeding during probing, gingival index, furcation involvement, loss of clinical adhesion and alveolar bone resorption were lower.^27^ On the other hand, conflicting results have been reported by Siasi Torbati et al,^28^ according to whom there was no association between the presence of the rs7975232 polymorphism in the vitamin D receptor gene and the development of periodontitis in the studied samples. Antonoglou et al^29^ reported no correlation between D binding protein in plasma and periodontal indices in patients with generalized aggressive periodontitis. Differences across studies may be influenced by ethnicity, genetic background, disease type and severity of periodontal disease.

 The present study found no significant association between the mean serum levels of omega-3 fatty acids and the severity of periodontitis. In contrast, a recent meta-analysis revealed that omega-3 supplementation may be associated with improvements in clinical parameters, including reductions in PD and CAL, when used as an adjunct to nonsurgical periodontal therapy.^15^ Additionally, another study by Castro Dos Santos et al^30^ indicated that in patients with periodontitis, the use of omega-3 fatty acid dietary supplements as supplements for nonsurgical periodontal treatment was associated with greater improvements in CAL and PD compared with nonsurgical treatment alone. Elgendy and Kazem^31^ also indicated that omega-3 supplements with SRP were associated with reduced periodontal inflammation and enhanced antioxidant status in postmenopausal women.Biologically, omega-3 fatty acids are believed to exert anti-inflammatory effects by modulating eicosanoid metabolism and promoting the production of specialized pro-resolving mediators (resolvins, protectins, and maresins), which help resolve inflammation and support tissue healing.^32,33^ The discrepancy between our findings and these intervention studies may reflect differences in dietary intake, bioavailability, study design, or sample size, as well as the cross-sectional nature of our study, which precludes causal conclusions.

## Limitations of the study

 One of the limitations of the current study was that, although periodontitis is a chronic disease that causes long-term pain, the study design was cross-sectional, meaning that vitamin measurements were conducted at a single point in time rather than periodically. The levels of some vitamins (for example, vitamin D) vary in different seasons due to different weather conditions. Additionally, the type of diet consumed at the time of measurement is also a factor in this issue, which necessitates adjustments for these confounding variables. Another limitation of the study is that the findings may not be generalizable to all populations due to the small sample size and the limited diversity of the study participants in terms of age and sex. Moreover, the study did not assess dietary intake or supplement use, which prevented an evaluation of the relative contribution of diet versus endogenous or supplemental sources to serum vitamin and omega-3 levels.

## Conclusion

 The present study showed that the serum levels of vitamins E and D were positively associated with periodontal status, whereas no significant association was observed for omega-3 fatty acids. It can be concluded that the optimal consumption of vitamins E and D, either as supplements or as part of an individual’s daily diet, may contribute to maintaining periodontal health. Also, the lack of significant findings for omega-3 in our study may be related to dietary intake variability, differences in metabolic pathways, or the limited sample size. Further longitudinal and interventional studies are required to clarify the potential preventive or therapeutic roles of these nutrients in periodontitis.

## Competing Interests

 Authors declare no conflicts of interest.

## Ethical Approval

 The Ethics Committee of Tabriz University of Medical Sciences approved the present study with the approval code of IR.TBZMED.REC.1402.134. In this study, the safety and health of the participants were observed. All the patients were informed about the objectives, benefits, and risks of participating in the study and signed an informed consent form before participating in the study. The patients were able to withdraw from the study at any time. Additionally, in this research, periodontitis was treated in patients.
